# The effects of adding zoledronic acid to neoadjuvant chemotherapy on tumour response: exploratory evidence for direct anti-tumour activity in breast cancer

**DOI:** 10.1038/sj.bjc.6605604

**Published:** 2010-03-16

**Authors:** R E Coleman, M C Winter, D Cameron, R Bell, D Dodwell, M M Keane, M Gil, D Ritchie, J L Passos-Coelho, D Wheatley, R Burkinshaw, S J Marshall, H Thorpe

**Affiliations:** 1 Academic Unit of Clinical Oncology, Weston Park Hospital, University of Sheffield, Sheffield, UK; 2 St James’ Institute of Oncology, University of Leeds, Leeds, UK; 3 Andrew Love Cancer Centre, Geelong, Victoria, Australia; 4 St James’ Institute of Oncology, Leeds Teaching Hospitals NHS Trust, Leeds, UK; 5 University Hospital Galway, Galway, Ireland; 6 Institut Català d’Oncologia, IDIBELL, L’Hospitalet de Llobregat, Barcelona, Spain; 7 Beatson Oncology Centre, Glasgow, UK; 8 Instituto Portugues de Oncologia, Lisboa, Portugal; 9 Royal Cornwall Hospital, Cornwall, UK; 10 Clinical Trials Research Unit, University of Leeds, Leeds, UK

**Keywords:** anti-tumour activity, breast cancer, chemotherapy, neoadjuvant, pathological response, ZOL

## Abstract

**Background:**

Pre-clinical studies have demonstrated synergistic anti-tumour effects of chemotherapy (CT) and zoledronic acid (ZOL). Within the AZURE trial, designed to determine whether the addition of ZOL to neoadjuvant therapy improves disease outcomes, a subgroup received neoadjuvant CT. We report a retrospective evaluation comparing pathological response in the primary tumour between treatment groups.

**Methods:**

In total, 205 patients received neoadjuvant CT±ZOL (CT+ZOL, *n*=102; CT, *n*=103). The primary end point was pathologically assessed residual invasive tumour size (RITS) at surgery. Secondary end points were pathological complete response (pCR) rate and axillary nodal involvement. Following review of surgical pathology reports (*n*=195), outcome differences between groups were assessed adjusting for potential response modifiers.

**Results:**

Baseline characteristics and CT treatments were similar. In multivariate analysis, allowing for biological and clinical factors known to influence tumour response, the adjusted mean RITS in CT and CT+ZOL groups were 27.4 and 15.5 mm, respectively, giving a difference in means of 12 mm (95% confidence interval: 3.5–20.4 mm; *P*=0.006). The pCR rate was 6.9% in the CT group and 11.7% in the CT+ZOL group (*P*=0.146). There was no difference in axillary nodal involvement (*P*=0.6315).

**Conclusion:**

These data suggest a possible direct anti-tumour effect of ZOL in combination with CT, warranting formal evaluation in prospective studies.

## Background

Zoledronic acid (ZOL), a nitrogen-containing bisphosphonate (N-BP), is firmly established in the management of metastatic bone disease. It inhibits farnesyl diphosphate synthase within the mevalonate pathway and, through this mechanism, is a potent inhibitor of osteoclast-mediated bone resorption. In addition, there are pre-clinical data indicating that farnesyl diphosphate synthase inhibition by ZOL has both direct and indirect anti-tumour effects in breast cancer (Winter *et al*, 2008). Furthermore, synergistic anti-tumour effects with chemotherapy (CT) drugs commonly used in the treatment of breast cancer have been demonstrated. Recently, it has been reported that sequential treatment with doxorubicin followed by ZOL at clinically relevant doses elicited substantial anti-tumour effects in *in vivo* mouse models of subcutaneous breast tumours (Ottewell *et al*, 2008). The clinical evidence of an anti-tumour effect of ZOL, however, is uncertain, although recent data suggest that the addition of ZOL to endocrine treatments improves disease outcomes in pre-menopausal women with early breast cancer (Gnant *et al*, 2009).

Inconclusive results from adjuvant trials of the oral bisphosphonate, clodronate, in the 1990s formed the rationale for adjuvant trials of the more potent ZOL. It was anticipated that ZOL might have more definite beneficial anti-tumour effects. This could occur ‘indirectly’ through the inhibition of bone resorption and consequent reduction in bone-derived factors that disrupt the inter-relationships between cancer, bone and haematopoetic stem cell populations, thereby creating a less favourable microenvironment for the survival of metastatic tumour cells. Alternatively, or perhaps in addition, ‘direct’ effects such as induction of tumour cell apoptosis, reduction in proliferation rates and tumour angiogenesis, as well as potential synergistic effects with anti-cancer therapies, might be clinically important (Coleman, 2007).

**Figure 1:**
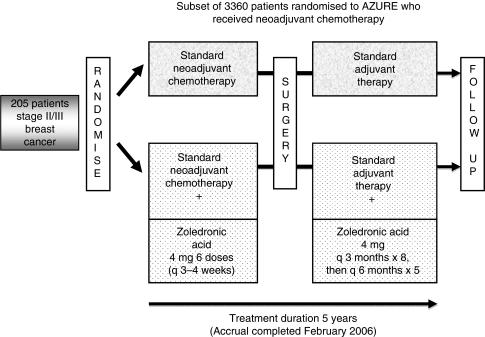
AZURE trial schema. (Does adjuvant zoledronic acid reduce recurrence in patients with high-risk localised breast cancer?)

The AZURE trial (ISRCTN 79831382; BIG 01/04) is an academic study run by the National Institute for Health Research National Cancer Research Network (NIHR NCRN) in the United Kingdom with input from international collaborative groups. The study randomised 3360 women with axillary node-positive stage II/III breast cancers to determine whether the addition of ZOL to systemic therapy improves disease-related outcomes (Figure 1). Analysis and reporting of the AZURE trial awaits the occurrence of a pre-specified number of disease-free survival events. However, within this large study, ∼6% of patients received neoadjuvant CT (Figure 1). In light of the pre-clinical data and emerging clinical evidence of a potential anti-tumour effect of ZOL, we have conducted an exploratory retrospective evaluation of this subgroup. The objective was to determine whether the addition of ZOL to primary systemic neoadjuvant CT had any demonstrable influence on pathological response in the surgical resection specimen compared with the effects of neoadjuvant CT alone.

## Patients and methods

### Patient population

Of the 3360 patients recruited, the treating physicians elected to treat 205 patients with neoadjuvant CT. Patients receiving neoadjuvant CT on the AZURE trial were required to have either stage T3 or T4 disease, or biopsy-proven lymph node involvement (N1), and be scheduled to proceed to definitive surgery and/or radical radiotherapy with curative intent within 6 months of starting therapy.

Using a minimisation technique to randomise patients, the two groups were well balanced for T-stage, lymph node involvement, oestrogen receptor (ER) status, neoadjuvant systemic treatment plan and CT type (anthracycline/taxane), menopausal status, the use of statins (which also act on the mevalonate pathway) and treatment centre.

Baseline tumour size *per se* was not prospectively collected in AZURE. Furthermore, inherent problems exist related to inter-observer variability in the clinical assessment of breast tumour size and discrepancies between physical examination, mammographic and ultrasound estimates of size. We therefore instead incorporated tumour (T) stage, determined by clinical examination, into the statistical analysis, as this was a prospectively collected tumour characteristic. However, we also sought information from centres on baseline tumour measurements by clinical, ultrasound and/or mammographic assessment to enable sensitivity analyses.

### Details of surgery and pathological response

Histopathologically assessed residual invasive tumour size (RITS) is reported on breast cancer pathology reports as part of the minimum data set, and the largest dimension of dominant invasive tumour focus is routinely recorded (NHS Breast Cancer Screening Programme (NHSBSP) and The Royal College of Pathologists, 2005). These data and the number of positive axillary lymph nodes at the time of definitive surgery were prospectively specified to be collected in AZURE, enabling the primary and secondary end points of this analysis to be determined.

The conventional end point of neoadjuvant CT studies in breast cancer is pathological complete response (pCR), representing the most robust surrogate marker of longer-term outcome (Fisher *et al*, 1997, 1998; van der Hage *et al*, 2001; Heys *et al*, 2002; Bear *et al*, 2006; Mieog *et al*, 2007). However, the definition of this has not been applied in a consistent and standardised manner throughout clinical trials (Kuroi *et al*, 2005). As AZURE was not primarily a neoadjuvant study, data regarding attainment of pCR were not prospectively collected. However, following a blinded central review of pathology reports, pCR was considered to have been achieved if the pathology report specifically indicated an absence of any residual invasive tumour within the breast and axilla, as per the recommended optimum definition (Gralow *et al*, 2008).

### Determining RITS and nodal status

In cases in which there was no residual invasive tumour, ‘0 mm’ was recorded. In cases in which multifocal pathology was present (*n*=25), the largest dimension of residual invasive tumour focus was recorded. Occasionally, if a measurement of the largest focus was not given, but the overall size including invasive tumour islands was reported, this was recorded.

For the assessment of axillary nodal disease after CT, the number of positive axillary nodes (and total number of nodes resected) was recorded. No distinction between axillary nodal micrometastases (>0.2–<2 mm) and overt metastases (⩾2 mm) was made, with both considered as evidence of nodal metastasis.

### Treatment

Case report forms were also evaluated to review details of neoadjuvant systemic treatments received. Duration of treatment was defined as the time from the first CT to surgery in days. Patients received neoadjuvant CT as per local guidelines. In light of the pre-clinical data, it was mandated that ZOL should be given after CT. Patients received ZOL as a 4 mg intravenous dose, given in 100 ml 0.9% normal saline over 15 min.

### Statistical methodology

The primary end point for this study was RITS (mm) in the surgical resection specimen. Secondary end points included the number of positive axillary nodes (categorised as 0, 1–3, 4+ positive nodes) and pCR (%), as previously defined. Only pre-defined end points were subjected to statistical testing using a 5% (two-sided) significance level. All other data were summarised descriptively. As this was an exploratory evaluation, patients were analysed on a per protocol basis, with one patient randomised to CT+ZOL but who never started study treatment included in the CT control arm.

For the primary end point, the outcome difference between the two groups was compared using linear regression, adjusting for the following potential prognostic factors: tumour stage (T2, T3 or T4, at the time of randomisation), ER status, PgR status, menopausal status, CT type (anthracycline, taxane) and treatment duration. Patients with missing data for PgR status and treatment duration (*n*=8) were included as ‘unknown’ PgR status and ‘6 months’, respectively, representing the maximum time between the commencement of neoadjuvant therapy and definitive surgery allowed by the study protocol. A sensitivity analysis was also performed that adjusted for clinical baseline tumour size, instead of tumour stage, and the other factors as above.

Axillary nodal status was compared between the two treatment groups using ordered logistic regression to adjust for the factors as described above. The difference between treatment groups in the proportion of patients with pCR was compared using logistic regression, adjusting for the previously described factors. Missing data for PgR status and treatment duration (*n*=8) were substituted as above.

HER2/neu status was not routinely assessed at the time of trial recruitment. Furthermore, very few patients received trastuzumab in the neoadjuvant treatment period (*n*=6). Tumour grade was not included in multivariate analysis, as grade, in most UK centres, is not routinely assessed on diagnostic core specimens because of concerns regarding possible tumour heterogeneity and sampling error. It is also difficult to evaluate post-treatment grade reliably because of CT-induced cytological and histological changes (Kurosumi, 2006).

### Role of the funding source

The trial design was reviewed and approved by Cancer Research UK's Clinical Trials Awards and Advisory Committee for access to NIHR NCRN resources. The research costs were supported by a grant from Novartis to the University of Sheffield, the sponsor of the study. The funders had no part in data collection and analysis, interpretation of data or writing the paper. The corresponding author had full access to the data and final responsibility for the decision to submit for publication.

## Results

Of the 205 patients who received neoadjuvant CT, 103 patients were randomised to CT alone and 102 to CT+ZOL. Of these, 195 (95.1%) surgical histopathology forms were available for central review, blinded to treatment allocation, and were included in these exploratory analyses; 94 received CT+ZOL and 101 received CT alone. Ten patients were excluded because of missing pathology reports (*n*=8), no surgery (*n*=1) or patient death before surgery (*n*=1).

Clinicopathological baseline characteristics are shown in Table 1. Age and menopausal status of patients in both groups were similar, although there were 10 more pre-menopausal patients in the CT-alone group. The majority of patients presented with T3 or T4 tumours and most had unknown lymph node involvement before the commencement of CT. Table 1 also shows median and interquartile range (IQR) for baseline tumour size by clinical, ultrasound and/or mammographic assessment where available. The frequency of ER-negative tumours, an important predictor of CT response (MacGrogan *et al*, 1996; Kaufmann *et al*, 2007), was similar in the groups. In approximately one-third of patients, PgR status was unknown (either missing or not performed in some recruiting centres). This trial began before the routine use of trastuzumab in adjuvant therapy and HER2 status was unknown in 32.3% of patients.

Details of systemic treatment received are shown in Table 2. Treatment duration and the number of CT cycles were comparable in both groups. Dose intensities were similar between the two treatment groups (data not shown).

### Residual invasive tumour size

Residual invasive tumour size could be determined in 182 patients (93 CT alone and 89 CT+ZOL). In the remaining 13 patients, 1 patient developed progressive disease during neoadjuvant CT and had palliative surgery almost 1 year later. For the remaining 12 patients (*n*=8 CT, *n*=4 CT+Z), the pathology reports gave no specific measurement of residual microscopic invasive tumour size describing ‘widespread tumour emboli’ or ‘occasional residual focus of infiltrating carcinoma’. Putting a measurement on these cases was not considered valid.

Median RITS was 30 mm (IQR 7–60 mm) in the CT-alone group and 21 mm (7–38 mm) in the CT+ZOL treatment group. In multivariate analysis (Table 3), the adjusted mean RITS in the CT-alone group was 27.4 and 15.5 mm in the CT+ZOL group, giving a statistically significant difference in means of 12 mm (95% CI 3.5–20.4 mm, *P*=0.0059). In a sensitivity analysis replacing T-stage with clinical tumour size at baseline, RITS remained smaller in the CT+ZOL group by 9.9 mm (95% confidence interval (CI) 0.2–19.7 mm; *P*=0.0465, *n*=137).

Multivariate analysis (Table 3) also revealed that treatment duration (*P*=0.0011) and taxane-based CT (*P*=0.0204) were significantly associated with RITS, indicating a smaller residual tumour size with increasing treatment duration and an increased residual tumour size with the use of taxane CT. This may, however, simply reflect an increased use of taxanes in patients responding poorly to the early cycles of anthracycline CT, as 31 patients planned at the time of randomisation to receive anthracyclines alone were subsequently switched to a taxane.

### Secondary end points

#### Axillary nodal status

Information on the pathological status of axillary lymph nodes was available from 194 (99.5%) pathology reports. The median number of positive axillary lymph nodes was 3 (IQR 0–6) in the CT group and 2 (IQR 0–6) in the CT+ZOL group. There was no significant difference in axillary nodal involvement, categorised by 0 (30.7% CT alone *vs* 28.7% CT+ZOL), 1–3 (23.8 *vs* 29.8%) or ⩾4 (45.5 *vs* 40.4%) positive nodes, between the treatment groups in multivariate analysis (*P*=0.6315). The only factors that were significantly associated with a lower number of involved nodes were increasing treatment duration (*P*=0.0102) and ER-negative status (*P*=0.0129).

#### pCR

In total, 24 (12.3%) patients had no residual invasive disease in the breast, 10 (9.9%) patients in the CT-alone group and 14 (14.9%) patients in the CT+ZOL group. In all, 18 (9.2%) patients achieved pCR, defined as ‘the absence of residual invasive tumour within both breast and axilla’, 7 (6.9%) patients in the CT-alone group and 11 (11.7%) in the CT+ZOL group. Of the patients achieving pCR, two in the CT+ZOL and none in the CT groups had DCIS present in the absence of any remaining invasive disease. In multivariate analysis (*n*=195) adjusting for potential prognostic factors in addition to neoadjuvant treatment group, a trend for patients receiving CT+ZOL to have increased odds of achieving pCR (odds ratio=2.2, 95% CI 0.8–6.3, *P*=0.1457) was seen. Not surprisingly, given the low number achieving pCR, no statistically significant predictive factors for pCR were identified.

#### Mastectomy rate

Breast conservation surgery is often the primary aim in patients being treated with neoadjuvant CT, and therefore mastectomy rate is an important outcome parameter. Initial (as a representation of intent of surgery) type of surgery was recorded in all patients. Altogether, 80 (79.2%) patients in the CT-alone group had a mastectomy compared with 66 (70.2%) in the CT+ZOL treatment group.

### Safety

The combination of CT+ZOL in the neoadjuvant treatment setting was well tolerated, with no increase in serious adverse event (SAE) reporting. There were 63 SAEs reported within the median neoadjuvant treatment period (147 days (IQR 129–184)): 36 events in the CT-alone group – 16 (44.4%) neutropenic sepsis, 7 (19.4%) neutropenia, 5 gastrointestinal, 3 pyrexia, 1 ‘other’ infection, 1 rash, 1 pleural effusion, 1 flu-like symptoms and 1 other symptom – and 27 in the CT+ZOL group – 14 (51.9%) neutropenic sepsis, 3 (11.1%) neutropenia, 3 (11.1%) ‘other’ infection, 5 gastrointestinal, 1 anaphylactic reaction and 1 cardiovascular or loss of consciousness.

## Discussion

These retrospective exploratory data intriguingly suggest that a bisphosphonate may have effects on tumours outside bone. An improved pathological response in patients treated with CT+ZOL compared with CT alone was observed and suggests a possible direct anti-tumour effect of ZOL in combination with neoadjuvant CT.

There are some limitations to the interpretation of these data. Firstly, there was no central pathological review of the operative specimens and therefore probable inter-pathologist variation existed with respect to specimen processing and pathological evaluation. Secondly, assessment of RITS is somewhat imprecise, as asymmetry of residual disease and hypocellularity are not usually quantified during pathology reporting (Symmans *et al*, 2007). In addition, missing data may negatively affect the quality of a retrospective study and, as exploratory end points, these analyses are likely to be underpowered.

In this study, we have included T-stage to reflect the baseline extent of primary breast cancer in the main analysis. Baseline clinical measurements of tumour size were missing in 26% of patients. In a sensitivity analysis, the replacement of T-stage with clinical estimates of initial tumour size into the multivariate analysis did not materially affect the differences in RITS seen between treatments, with the beneficial effect of ZOL persisting.

The extent of residual tumour following CT has been well established as an intermediate surrogate end point for longer-term outcome such as relapse and survival (Carey *et al*, 2005). However, no standard validated internationally accepted classification exists for the reporting of pathological response to neoadjuvant CT. To date, neoadjuvant studies have used pCR as a validated intermediate surrogate marker of survival, despite it being a less common phenomenon. Therefore, for the majority of patients, ‘residual disease’ represents a spectrum ranging from those patients achieving ‘near’ pCR to those with resistant disease, potentially associated with widely differing prognoses.

Analysis of RITS potentially allows an exploratory evaluation of therapeutic effect in those patients achieving less than pCR. Furthermore, RITS after CT is of potential clinical relevance. In the largest published single-institution cohort from the MD Anderson Cancer Center (*n*=338), Chen *et al* (2004) published data on 5-year locoregional recurrence (LRR) rates following neoadjuvant CT and breast-conserving surgery. Pathologically assessed residual primary tumour >2 cm after neoadjuvant CT and breast-conserving surgery significantly predicted for LRR in multivariate analysis (hazard ratio 3.2; *P*=0.006, along with initial N2/N3 disease, residual multifocal pattern of disease and lymphovascular space invasion).

Although not widely validated, published studies have shown that pathological staging, including an evaluation of residual tumour size after neoadjuvant CT, may be useful in predicting longer-term outcome. In 2003, a revised AJCC TNM breast cancer classification was implemented (Greene *et al*, 2002), designating the letter ‘y’ to represent pathological stage of residual tumour after neoadjuvant therapy. Carey *et al* (2005) reported that higher pathological stage in the surgical resection specimen using this revised classification system was significantly associated with lower rates of distant disease-free survival and overall survival in stage II/III breast cancer patients treated with neoadjuvant CT (*n*=132, median follow-up 5 years). In addition, the Clinical-Pathologic Scoring (CPS) system has very recently been described to predict 5-year outcomes of breast cancer patients treated with neoadjuvant CT (Jeruss *et al*, 2008). This model incorporates clinical TNM stage at presentation and post-neoadjuvant CT pathological TNM stage, assigning points to each presenting clinical substage and post-treatment pathological substage, with a 5-year distant metastasis-free survival of 97% for a CPS score of 0 and 46% for a CPS score of 4.

In this analysis, we have reported a trend for a higher proportion of patients treated with CT+ZOL (11.7%) achieving pCR compared with CT alone (6.9%). It is not surprising that this is not statistically significant because of the small numbers of patients involved. Secondly, these figures require a degree of cautious interpretation, as they represent overall low rates of pCR, given that pCR rates of up to 30% have been reported in randomised studies of neoadjuvant CT regimens (Buzdar, 2007). However, it must be noted that many of the previously published studies included patients with T1–T2 stage disease, whereas in this cohort 98% of patients presented with T3–T4 disease.

The presence of nodal disease after neoadjuvant CT is associated with a poorer prognosis (Kuerer *et al*, 1999; Rouzier *et al*, 2002). We did not show any significant difference in post-treatment nodal status between the two treatment groups. This may not be surprising given that axillary lymph node metastases, considered as disseminated tumour cells, are selectively more resistant than primary tumour cells (von Minckwitz *et al*, 2008). However, of interest, the proportion of patients in the CT+ZOL group achieving pCR (including negative nodal status) was higher than in the CT-alone group, potentially reflecting increased chemosensitivity of nodal disease with complete pathological response in the breast, as has been previously described (Kuerer *et al*, 1999).

These data reported here follow shortly after the most promising clinical data to date regarding a potential anti-tumour effect of ZOL in early breast cancer from the ABCSG-12 study report (Gnant *et al*, 2009). In this trial, 1803 pre-menopausal women with endocrine-sensitive breast cancer were randomised to receive endocrine treatment (gonadotrophin-releasing hormone analogue and tamoxifen or anastrozole) with or without ZOL (4 mg intravenous infusion) 6 monthly for 3 years. With a median follow-up of 48 months, the addition of ZOL to endocrine therapy was associated with a 36% improvement in disease-free survival compared with endocrine treatment alone (hazard ratio=0.64 95% CI 0.46, 0.91, *P*=0.01). Furthermore, the benefits of ZOL were not confined to the bone, with a striking reduction in locoregional and distant extraosseus recurrence events as well. Despite a relatively low number of events, this is further evidence that ZOL may exhibit anti-tumour activity in patients with early breast cancer.

In the clinical setting, there are no fully published data regarding the potential anti-tumour effect of ZOL in combination with CT. However, there are pre-clinical data from several different *in vitro* and *in vivo* model systems of the beneficial anti-tumour effects of combination therapy with anti-cancer agents and ZOL (Santini *et al*, 2006). More specifically to breast cancer, beneficial effects of the use of combination treatments including ZOL and cytotoxic therapies have been shown in *in vivo* model systems of breast cancer, most of which included a high degree of tumour-induced bone disease. However, Ottewell *et al* (2008) recently reported that sequential treatment with clinically relevant doses of doxorubicin, followed 24 h later by ZOL, inhibited subcutaneous breast tumour growth in a mouse model of breast cancer soft tissue disease in the absence of tumour bone disease. The two drugs alone or in the reverse sequence had little or no effect on tumour growth. Simultaneous administration of doxorubicin and ZOL also reduced tumour growth somewhat, but only when the two drugs were given in the sequence of doxorubicin followed by ZOL was complete and durable inhibition of tumour growth achieved. The reduction in subcutaneous tumour growth was accompanied by increased tumour cell apoptosis, reduction in tumour cell proliferation and tumour angiogenesis. The molecular mechanisms behind this sequence-dependent synergy remain to be established, but giving sequenced treatment elicited changes in the expression of a number of genes and proteins associated with cell-cycle progression and apoptosis not occurring with single-agent doxorubicin or ZOL (Ottewell *et al*, 2010). These data therefore suggest that low serum concentrations of ZOL, in combination with cytotoxic drugs, are sufficient to exert anti-tumour effects in peripheral tissues and lead to the hypothesis that there may be beneficial anti-tumour effects in patients with early breast cancer.

In summary, these are the first clinical data suggesting that the addition of ZOL to neoadjuvant CT may improve pathological response in breast cancer, and give further support to other emerging data that ZOL may exhibit anti-tumour activity in combination with anti-cancer therapies. Formal evaluation in prospective neoadjuvant randomised trials incorporating detailed assessment of biomarkers is recommended.

## Figures and Tables

**Table 1:** Patient characteristics

	**Chemotherapy alone *n*=101 (%)**	**Chemotherapy+ zoledronic acid *n*=94 (%)**
Median age in years (IQR)	48 (41–54)	47 (42–57)
		
*T-stage (at time of randomisation)*		
T2	1 (1)	3 (3.2)
T3	55 (54.5)	54 (57.4)
T4	45 (44.6)	37 (39.4)
		
*Axillary lymph involvement (at time of randomisation)*		
1–3	6 (5.9)	6 (6.4)
Unknown	95 (94.1)	88 (93.6)
		
*Menopausal status*		
Pre	65 (64.4)	55 (58.5)
Post	28 (27.7)	31 (33)
Unknown	8 (7.9)	8 (8.5)
		
*ER status*		
+ve	65 (64.4)	64 (68.1)
−ve	36 (35.6)	30 (31.9)
		
*PgR status*		
+ve	25 (24.8)	32 (34)
−ve	46 (45.5)	33 (35.1)
Unknown	30 (29.7)	29 (30.9)
		
*HER2 status*		
+ve	22 (21.8)	14 (14.9)
−ve	47 (46.5)	49 (52.1)
Not measured	26 (25.7)	24 (25.5)
Unknown	6 (5.9)	7 (7.4)
		
*Histology*		
Ductal	78 (77.2)	75 (79.8)
Lobular	12 (11.9)	7 (7.4)
Other	7 (6.9)	10 (10.6)
Unknown	4 (4)	2 (2.1)
		
Median number of lymph nodes examined (IQR)	15 (11–19)	14 (11–19)
		
*Median tumour size in mm (IQR)*		
Clinical (*n*=144–74%)	70 (60–100)	60 (40–70)
Ultrasound (*n*=91–47%)	32 (25–46)	31 (26–40)
Mammogram (*n*=67–34%)	40 (30–60)	35 (27–41)

Abbreviations: ER=oestrogen receptor; IQR=interquartile range; PgR=progesterone receptor.

**Table 2:** Neoadjuvant chemotherapy treatments

	**Chemotherapy alone *n*=101 (%)**	**Chemotherapy +zoledronic acid *n*=94 (%)**
Median duration of treatment days, (IQR)	145.5 (127.5–184)	148 (130–188)
Median number of chemotherapy cycles (IQR)	6 (6–7)	6 (6–8)
		
*Chemotherapy received*		
Anthracycline based only	46 (45.5)	49 (52.1)
Sequential anthracycline+taxane	50 (49.5)	41 (43.6)
Combination anthracycline+taxane	5 (5)	4 (4.3)
Trastuzumab	4 (4)	2 (2.1)
G-CSF use	18 (17.8)	22 (23.4)
		
Median number of zoledronic acid infusions (IQR)	N/A	6 (5–6)
Median number of zoledronic acid infusions given with chemotherapy (IQR)	N/A	5 (4–6)

Abbreviations: G-CSF=granulocyte colony-stimulating factor; IQR=interquartile range; N/A=not applicable.

**Table 3:** Multivariate analysis investigating residual invasive tumour size by neoadjuvant treatment (adjusting for prognostic factors)

**Factor**	**Estimate**	**s.e.**	**95% CI**	***P*-value**
*Treatment*				
CT alone *vs* **CT**+zoledronic acid	12	4.3	3.5, 20.4	0.0059
				
*T-stage*				
T2 *vs* **T4**	−20.7	14.7	−49.6, 8.3	0.2870
T3 *vs* **T4**	2	4.5	−6.8, 10.8	
				
*Taxane regimen*				
Yes *vs* **no**	11.9	5.1	1.9, 21.9	0.0204
				
*Treatment duration*				
a	−0.2	0.1	−0.3, −0.1	0.0011
				
*ER status*				
Positive *vs* **negative**	10.2	5.4	−0.5, 21	0.0609
				
*PgR status*				
Positive *vs* **negative**	0.4	6.2	−11.9, 12.7	0.1466
Unknown *vs* **negative**	−9.2	5.7	−20.5, 2.1	
				
*Menopausal status*				
Pre *vs* **post**	−9.8	4.8	−19.3, −0.2	0.0567
Unknown *vs* **post**	−16.5	8.3	−32.9, −0.1	

Abbreviations: CI=confidence interval; CT=chemotherapy; ER=oestrogen receptor.

a Defined as time from the first chemotherapy to surgery (days); a negative estimate indicates tumour size decreasing with increasing treatment duration.

Reference categories are shown in bold.

A positive estimate indicates that the residual invasive tumour size is decreased in the reference category.
